# The Fungus *Tremella mesenterica* Encodes the Longest Metallothionein Currently Known: Gene, Protein and Metal Binding Characterization

**DOI:** 10.1371/journal.pone.0148651

**Published:** 2016-02-16

**Authors:** Paul Iturbe-Espinoza, Selene Gil-Moreno, Weiyu Lin, Sara Calatayud, Òscar Palacios, Mercè Capdevila, Sílvia Atrian

**Affiliations:** 1 Departament de Genètica, Facultat de Biologia, Universitat de Barcelona, Barcelona, Spain; 2 Departament de Química, Facultat de Ciències, Universitat Autònoma de Barcelona, Cerdanyola del Vallès, Barcelona, Spain; Yonsei University, REPUBLIC OF KOREA

## Abstract

Fungal Cu-thioneins, and among them, the paradigmatic *Neurospora crassa* metallothionein (MT) (26 residues), were once considered as the shortest MTs -the ubiquitous, versatile metal-binding proteins- among all organisms, and thus representatives of their primeval forms. Nowadays, fungal MTs of diverse lengths and sequence features are known, following the huge heterogeneity of the Kingdom of Fungi. At the opposite end of *N*. *crassa* MT, the recently reported *Cryptococcus neoformans* CnMT1 and CnMT2 (122 and 186 aa) constitute the longest reported fungal MTs, having been identified as virulence factors of this pathogen. CnMTs are high-capacity Cu-thioneins that appear to be built by tandem amplification of a basic unit, a 7-Cys segment homologous to *N*. *crassa* MT. Here, we report the *in silico*, *in vivo* and *in vitro* study of a still longer fungal MT, belonging to *Tremella mesenterica* (TmMT), a saprophytic ascomycete. The *TmMT* gene has 10 exons, and it yields a 779-bp mature transcript that encodes a 257 residue-long protein. This MT is also built by repeated fragments, but of variable number of Cys: six units of the 7-Cys building blocks-CXCX_3_CSCPPGXCXCAXCP-, two fragments of six Cys, plus three Cys at the N-terminus. TmMT metal binding abilities have been analyzed through the spectrophotometric and spectrometric characterization of its recombinant Zn-, Cd- and Cu-complexes. Results allow it to be unambiguous classified as a Cu-thionein, also of extraordinary coordinating capacity. According to this feature, when the TmMT cDNA is expressed in MT-devoid yeast cells, it is capable of restoring a high Cu tolerance level. Since it is not obvious that *T*. *mesenterica* shares the same physiological needs for a high capacity Cu-binding protein with *C*. *neoformans*, the existence of this peculiar MT might be better explained on the basis of a possible role in Cu-handling for the Cu-enzymes responsible in lignin degradation pathways.

## Introduction

Metallothioneins (MTs) constitute a heterogeneous superfamily of ubiquitously occurring, low molecular weight, cysteine rich proteins that natively coordinate divalent (Zn^2+^, Cd^2+^) or monovalent (Cu^+^) metal ions through metal-thiolate bonds, which imposes a definite polypeptide folding (see [1,2,3] for recent MT reviews). No single biological role has been assigned to these peptides, but, instead, several functions have been proposed [4], ranging from physiological metal handling to toxic metal protection. MTs are highly polymorphic proteins exhibiting a low degree of sequence similarity, so that in fact they can be considered different homology groups along the Tree of Life [5]. It is precisely this sequence heterogeneity what explains why different classification criteria have emerged during the progressive discovery and characterization of new MTs. Binz and Kägi, in the late nineties [6], proposed a taxonomy-based MT classification, in which essentially each MT group (so-called *family*) included the MTs of a taxonomic group of organisms, this ensuring their homology. In contrast to this *sequence-based* criterion, we proposed a MTs *function-based* classification that assigns a Zn-/Cd (*i*.*e*. divalent metal ion) or Cu-thionein (*i*.*e*. monovalent metal ion) character to each peptide, according to its metal-binding preference [7], a classification that was latter modulated as a step gradation between these two extreme metal preferences [8,9]. Thereafter, we showed that a unique, energetically optimized complex results when a MT polypeptide folds about its cognate metal ions, while with non-cognate metal ions, it renders a mixture of species, none of them specially favored [10].

The Kingdom of Fungi is an extremely large and heterogeneous group of organisms, comprising between 1.5 million to 5 million species. This same heterogeneity applies to their MTs, because as for no other taxon fungal MTs are distributed in 6 different families (from Family #8 to #13) in the Binz and Kägi classification [6] (Table 1). At the time of such proposal, the fungal non-yeast MTs (Family #8) were restricted to those of the ascomycete *Neurospora crassa* [11] and the basidiomycete *Agaricus bisporus* (the edible champignon or white mushroom) [12]. Both were the shortest MTs reported (26 amino acids, encompassing 7 Cys residues), and shared with Cup1 (the yeast *Saccharomyces cerevisiae* MT [13,14]) its definite Cu-thionein character. From these results, the idea that fungal non-yeast MTs were representative of short, archetypical MTs, which would have evolved to yield the higher invertebrate and vertebrate forms by domain duplication and specialization towards divalent metal ion binding, gained full support [15].

**Table 1:** Families of the Binz & Kägi MT classification that include the fungal MTs.

Family	Group	Example	Sequence	UniProtKB
8	**Fungal 1**	*N*. *crassa* MT	MGDCGCSGASSCNCGSGCSCSNCGSK	P02807
9	**Fungal 2**	*C*. *glabrata* MT1	MANDCKCPNGCSCPNCANGGCQCGDKCECKKQSCHGCGEQCKCGSHGSSCHGSCGCGDKCECK	P15113
10	**Fungal 3**	*C*. *glabrata* MT2	MPEQVNCQYDCHCSNCACENTCNCCAKPACACTNSASNECSCQTCKCQTCKC	P15114
11	**Fungal 4**	*Y*. *lipolytica* MT3	MEFTTAMLGASLISTTSTQSKHNLVNNCCCSSSTSESSMPASCACTKCGCKTCKC	Q9HFD0
12	**Fungal 5**	*S*. *cerevisiae* Cup1	MFSELINFQNEGHECQCQCGSCKNNEQCQKSCSCPTGCNSDDKCPCGNKSEETKKSCCSGK	P0CX80
13	**Fungal 6**	*S*. *cerevisiae* Crs5	MTVKICDCEGECCKDSCHCGSTCLPSCSGGEKCKCDHSTGSPQCKSCGEKCKCETTCTCEKSKCNCEKC	P41902

This picture was completely turned over when the two MTs of the human pathogenic fungus *Cryptococcus neoformans* (CnMT1 and CnMT2) were recently reported as infection, virulence, and pathogenicity factors [16]. CnMT1 and CnMT2 counteract the Cu(I) ions diffused by macrophages in the infected tissues through the extraordinary Cu-binding capacity derived from their unusual length: 122 (CnMT1) and 183 amino acids (CnMT2) [17]. These two MTs revealed an unexpected modular structure, being respectively constituted by three and five 7-Cys regions separated by spacer stretches. Therefore, their origin was hypothesized to be the result of ancient tandem repetitions of a primeval fungal MT unit comprising seven Cys residues, with the same Cys pattern of the *Neurospora* and *Agaricus* MTs (X_2_-[CXC]-X_5_-[CXC]-X_3_-[CXC]-X_2_-C-X_3_) ([11,12], respectively) [16]. The analysis of the Cu-binding features of CnMT1 and CnMT2 further supported this hypothesis, because the homometallic Cu-CnMT species folded *in vivo* at high Cu concentrations could be readily explained by the respective three- and five-fold presence of basic Cu_5_-(7-Cys) clusters [17]. The other known “long MTs” are the five MT isoforms present in the ciliate *Tetrahymena* species, thus another unicellular eukaryote, with lengths ranging between 96 and 181 amino acids, most of them also with modular primary structures [18].

**Fig 1:**
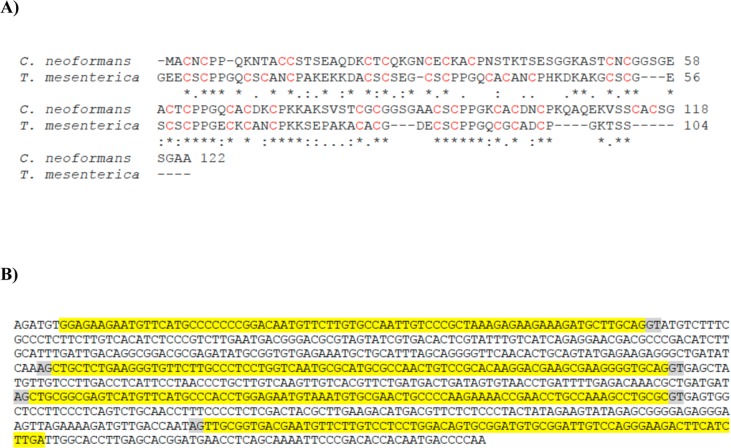
Partial *T*. *mesenterica* MT protein and cDNA sequences retrieved from data banks. (A) Clustal W2 alignment of the *C*. *neoformans* CnMT2 protein sequence and that retrieved from the *T*. *mesenterica* NCBI EIW70699 ORF annotation. (B) cDNA corresponding to the sequence coding for the protein shown in (A) localized in the TREMEscaffold_3 (access code JH711530.1) of the *T*. *mesenterica* genome. The putative exons are shown in yellow and the conventional splicing donor/acceptor sites in gray.

During the study of *C*. *neoformans CnMT1* and *CnMT2*, there was evidence that both genes had been wrongly annotated [17]. Miss annotation of MTs or MT-like’s often occurs as the result of automatic analysis of raw genome sequences, due to the common short exon length of the *MT* genes, and the Cys repetition in MT proteins. In this scenario, we aimed at examining the ensemble of available fungus genomes, transcript and EST databases, in order to identify other putative long-length MTs, and to analyze their putative modular structure and metal binding abilities. Among all the retrieved hits, a partial cDNA from *Tremella mesenterica*, devoid of translation starting codon, and annotated as coding a hypothetical protein (Fig 1) attracted our attention. This sequence showed a Cys pattern characteristic of MT polypeptides, it aligned well with the *C*. *neoformans* MTs, and it belonged to a fungus genus also member of the Tremellales order [19]. *T*. *mesenterica* is typically considered as a saprophytic fungus, but it has also been reported as parasitic to other fungi. Hence, starting from the partial and unassigned sequence, we were able to define, both bioinformatically and experimentally, the whole polypeptide coding sequence, showing that it had all the requirements to be considered as an MT protein, which in fact turned out to be the longest MT ever reported (257 amino acids), and it also exhibited a modular structure. The *T*. *mesenterica* MT (TmMT) was then recombinantly synthesized as Zn-, Cd- and Cu-complexes, which were spectroscopically and spectrometrically characterized. The results showed that TmMT was also a Cu-thionein, with an extremely high Cu coordination capacity. This is in concordance with the high tolerance exhibited by this fungus to Cu. Our results open the possibility to ascertain the biological significance that this protein may exhibit in the *T*. *mesenterica* physiology, but most significantly, they show how tandem amplification of basic MT units seem to be a common trait in the evolution of several MTs in the Tremellales fungi.

## Materials and Methods

### Bioinformatic methods for identification of MT genes in the *Tremella mesenterica* genome

DNA and protein sequences were retrieved, analyzed and compared using the online versions of BLAST (at www.ncbi.nim.nih.gov) and Clustal Omega2 (W2) (at http://www.ebi.ac.uk). Screened databases were NCBI (National Center for Biotechnology Information, at www.ncbi.nim.nih.gov) the JCI (Joint Genome Institute of the USA Department of Energy (at genomeportal.jgi.doe.gov) [20].

### *Tremella mesenterica* cultures

*Tremella mesenterica* strain was obtained from PYCC^®^ (Portuguese Yeast Culture Collection, ref # PYCC 5472). Liquid cultures were performed at 25°C in YM (Yeast Mold) medium (yeast extract 3 g/L, malt extract 3 g/L, peptone 5 g/L, and glucose 10 g/L) [21], supplemented with copper salt when necessary (CuSO_4_, at concentrations ranging from 0.1 mM to 5 mM, as indicated in each experiment). For plate cultures, YPD (2% glucose, 1% yeast extract, 2% peptone and 2% agar) was used and supplemented with CuSO_4_ when necessary.

### *Tremella mesenterica* RNA isolation and retrotranscription

Total RNA was extracted from *T*. *mesenterica* 10-mL YM cultures, following an adaptation of yeast RNA isolation rationale [22]. All the used material was sterilized and treated to be RNAase free. 1-mL aliquots of grown cultures were centrifuged in Eppendorf tubes for 3 min at 2500 rpm, frozen in liquid N_2_ and re-suspended in 0.5 mL of LETS buffer (0.1 mM LiCl, 0.01 mM EDTA, 0.01 mM Tris, 2% SDS, pH 4.3). Further 0.5 mL of Tris-HCl saturated phenol (pH 4.3) and 0.5 mL of glass beads (425–600 μm diameter) were added to each Eppendorf and cells were disrupted in a TissueLyser^®^ (Qiagen) by two 30-s series of 30 pulse/s. A mixture of phenol:chlorophorm:isoamyl alcohol (25:24:1) was added 1:1 v:v to the supernatant of a 15-min centrifugation at 12000 rpm for a first extraction. A second extraction was performed from the previous supernatant with 24:1 chlorophorm:isoamyl alcohol, and samples were precipitated with 5 M LiCl and kept at -80°C for at least 3 h. After centrifugation at 12000 rpm for 15 min, the pellet was washed with 200 μL of 70% ethanol per Eppendorf, and re-precipitated. Finally, the RNA was re-suspended in 30 μL of milliQ water per Eppendorf and its concentration assessed by A_260_ in a NanoQuant^®^ (Tecan) equipment, and by agarose gel electrophoresis (1% agarose in TAE (40 mM Tris, 20 mM acetic acid, 1 mM EDTA) buffer. The isolated RNA was treated with RQ1^®^ RNAse-Free DNAse (Promega) to avoid DNA contamination (digestion with 1 u/μL of the enzyme at 37°C for 30 min) and stored at -80°C until needed. Total RNA was retrotranscribed using the Transcriptor First Strand cDNA Synthesis^®^ kit (Roche), which includes oligo dT and random hexamer primers. 1 ng of total RNA was denatured for 5 min at 65°C, and then RT buffer (8 mM MgCl_2_), RNAse inhibitor, RT enzyme and the oligonucleotide mix were added in a 20-μL final volume. The sample was incubated for 10 min at 25°C, and retrotranscription was allowed for 30 min at 55°C and finally stopped by a 5-min incubation at 85°C. The obtained cDNA was quantified by A_260_ measurement in a NanoQuant^®^ (Tecan).

### RACE amplification of the *Tremella mesenterica* mRNA

To obtain the full *T*. *mesenterica* MT cDNA 5’ end sequence, the RACE (Rapid Amplification of cDNA Ends) strategy was applied, through the 5’ RACE v12^®^ kit from Roche. To this end, three antisense primers were designed from the already known TmMT sequence: R1 (5’ TCAAGATGAAGTCTTCCCTG 3’), R2 (5’ GACAATCCGCACATCCGCAC 3’) and R3 (5’ ACATTCGTCACCGCAAGCGC 3’). RACE reactions were performed following the supplier instructions, starting from 2 μg of the total *T*. *mesenterica* RNA preparation (147.5 μg RNA/μL) obtained from fresh fungus cultures in YM medium. The products of the nested PCR of the RACE steps were followed by agarose gel electrophoresis, and the final product was directly sequenced.

### Synthesis of the *Tremella mesenterica* cDNA and cloning in the *E*. *coli* expression vector

cDNA cloning procedures were performed essentially as described previously in detail for the *C*. *neoformans* [17] and *Amphioxus* [23] MTs. Hence, the complete TmMT cDNA was amplified by PCR from the total *T*. *mesenterica* cDNA preparation obtained as previously described. The reaction was performed in a final volume of 25 μL, using the Expand High Fidelity PCR system^®^ (Roche), and the specific primers: 5’ 5’AAAAGGATCCATGTCTGCTCCTGTCGAAAC 3’ (upstream) and 5’AAAACTCGAGGATTTGACGTTAGAGCAACC 3’ (downstream), to respectively add the *BamH*I/*Xho*I sites necessary for in-frame cloning into the *E*. *coli* pGEX-4T-1^®^ expression vector (GE Healthcare). Expression from this system yields GST-fusion proteins, from which the MT portion is isolated by thrombin cleavage [24]. The 35-cycle amplification reaction was performed under the following conditions: 15 s at 94°C (denaturation), 50 s at 55°C (annealing), and 50 s at 72°C (elongation). The final product was analyzed by 2% agarose gel electrophoresis/ethidium bromide staining, and directly purified from the PCR reaction with the Illustra DNA Purification Kit^®^ (GE Healthcare). The amplified DNA and the pGEX-4T-1 vector were digested with *BamH*I and *Xho*I (Fast Digest^®^, Thermo Scientific), and ligated using the DNA Ligation Kit 2.1^®^ (Takara Bio Inc.). Finally, the recombinant plasmids were transformed into *E*. *coli* Mach I strain for sequencing, which was attained using the Big Dye Terminator 3.1 Cycle Sequencing Kit^®^ (Applied Biosystems) and the pGEX-4T-1 5’ and 3’ primers, in a automatic sequencer (ABIPRISM 310, Applied Biosystems) of the CCiTUB (Genomics Services of the University of Barcelona). The correct recombinant plasmids (pGEX-TmMT) were then transformed into the *E*. *coli* BL21 protease deficient strain (GE Healthcare) for protein synthesis.

### Cloning of the *Tremella mesenterica* cDNA in yeast expression vector and complementation assays

The *Saccharomyces cerevisiae* 51.2cΔc5 strain (*MAT*a, *trp*1-1, *ura*3-52, *ade-*, *his-*, *CAN*^*R*^, *gal*1, *leu2-3*, *112 met13*, *cup1*Δ::*URA*3 *crs5*Δ::*LEU2*), derived from VC-sp6 [25] was used for copper tolerance assays as described in detail previously for the CnMT1-derived peptides [26]. The TmMT cDNA was inserted into the *BamH*I/*Xho*I sites of the yeast vector p424-GPD, which contains TRP1 as a selection marker, the constitutive glyceraldehyde-3-phosphate dehydrogenase (GPD) promoter for gene expression, and the cytochrome-c-oxidase (CYC1) transcriptional terminator [27]. The recombinant TmMT-p424 plasmid was transformed into 51.2cΔc5 cells using the LiAc/SS-DNA/PEG procedure [28], and positive transformants were selected by their capacity to grow in synthetic complete medium (SC) devoid of Trp, Leu, and Ura. For comparative purposes, yeast transformants with CUP1-p424 and mMT1-p424, encoding for the yeast CUP1 MT and the mouse MT1 isoform respectively, and with the void p424 plasmid, were also assayed. For copper tolerance tests, the transformants were initially grown in selective SC-Trp-Ura medium at 30°C and 220 rpm until saturation, and then a cell suspension of OD_600_ = 0.01 was used to inoculate 3-mL fresh cultures supplemented with CuSO_4_ at 0, 2, 4, 7, 10, 15, 20, and 30 μM final concentrations. After 18 h of growth, the final OD_600_ was measured and plotted as a percentage of the OD_600_ reached by the culture grown without Cu. Three replicates were run for each Cu concentration, and for each transformant.

### Preparation of recombinant and *in vitro*-constituted metal-TmMT complexes

5-L LB (Luria-Bertani) cultures of pGEX-TmMT transformed BL21 cells were induced with 100 μM (final concentration) of IPTG (isopropyl β-D-thiogalactopyranoside), and after 30-min growth, they were supplemented with 300 μM ZnCl_2_, 300 μM CdCl_2_ or 500 μM CuSO_4_ (final concentrations) to respectively allow the synthesis of the Zn-, Cd- or Cu-TmMT complexes, and further grown for additional 2.5 h. Cu-supplemented cultures were performed at two aeration conditions: regular (*i*.*e*. 1-L of LB media in a 2-L Erlenmeyer flask, at 250 rpm) or low (1.5-L of LB media in a 2-L Erlenmeyer flask, at 150 rpm), since available oxygen determines the intracellular copper levels in the host cells, as described in [29]. To prevent oxidation of the metal–TmMT complexes, argon was bubbled in all the steps of the purification protocol. Cells recovered from the bacterial cultures by centrifugation were resuspended in ice-cold PBS (1.4 M NaCl, 27 mM KCl, 101 mM Na_2_HPO_4_, 18 mM KH_2_PO_4_)-0.5% v/v β-mercaptoethanol and disrupted by sonication in a Sonifier^®^ ultrasonic cell disruptor (8 min, at 0.6 pulse/s). The total protein extract was centrifuged at 12,000 x*g*, 40 min, and the supernatant was incubated with Glutathione-Sepharose 4B^®^ (GE Healthcare) beads at gentle agitation for 1 h, room temperature. After three washes in cold PBS, the matrix-bound GST-MT protein was split by thrombin digestion (10 u per mg of fusion protein, overnight at 17°C). The solution containing the metal-TmMT complexes, which had consequently been released from the matrix, was concentrated using Ultracel^®^ YM-3 (Millipore) filters, and finally fractionated through a Superdex-75 FPLC column (GE Healthcare) equilibrated with 20 mM Tris-HCl, pH 7.0, and run at 0.8 mL min^-1^. The protein-containing fractions, identified by their absorbance at 254 and 280 nm, were later analyzed in 15% SDS-PAGE gels stained with Coomassie Blue, and they were pooled and stored at -80°C until further use. Due to the pGEX vector cloning specificities, the recombinant TmMT exhibited two additional N-term residues (Gly-Ser), but they have been shown to have no effect on the MT metal-binding features [30]. Further details about the synthesis and purification procedures can be found in previous publications [17,23,26].

The so-called “*in vitro* complexes” were those prepared *via* metal replacement by adding the corresponding metal ions (Cd(II) or Cu(I)) to the recombinant Zn-TmMT samples. These reactions were performed at pH 7.0 using CdCl_2_ or [Cu(CH_3_CN)_4_]ClO_4_ solutions, respectively, as described earlier in detail for mammalian MTs [24,31]. During all the experiments, strict oxygen-free conditions were maintained by saturating the solutions with argon. All the *in vitro*-obtained metal-MT samples were analyzed following the same rationale as for the recombinant samples.

### Characterization of the metal-TmMT complexes

ICP-AES (inductively coupled plasma atomic emission spectroscopy) analysis of the purified metal-TmMT complexes was essentially performed as previously described for other MTs [17,23,26]. S, Zn, Cd and Cu contents were measured in a Polyscan 61E (Thermo Jarrell Ash) spectrometer, reading S at 182.040 nm, Zn at 213.856 nm, Cd at 228.802 and Cu at 324.803 nm. Samples were treated as in [32], and were also alternatively incubated in 1 M HCl at 65°C for 15 min (acid ICP conditions) to eliminate any labile sulfide ions, as described in [33]. Protein concentration was calculated from the acid ICP-AES sulfur content, assuming that the measured S atoms were contributed by the MT peptide. CD spectra were recorded in a Jasco spectropolarimeter (model J-715) interfaced to a computer (J700 software) at a constant temperature of 25°C, maintained by a Peltier PTC-351S apparatus. Electronic absorption was measured in a HP-8453 Diode array UV–visible spectrophotometer. All spectra were recorded using 1-cm capped quartz cuvettes, corrected for the dilution effects and processed using the GRAMS 32 program.

### ESI-MS (electrospray ionization mass spectrometry) analyses of the metal-TmMT complexes

Electrospray ionization time-of-flight mass spectrometry (ESI-TOF MS) conditions for the analysis of the metal-TmMT complexes were adapted from those reported in detail in [17] and [26]. The equipment used was a Micro TOF-Q (Bruker) interfaced with a Series 1200 HPLC Agilent pump and an autosampler, all of them controlled by the Compass Software. The mass spectrophotometer was calibrated with ESI-L Low Concentration Tuning Mix (Agilent Technologies). The Zn- and Cd-TmMT samples were analyzed under the following conditions: 20 μL of protein solution injected through PEEK (polyether heteroketone) tubing (1.5 m x 0.18 mm i.d.) at 40 μL min^-1^; capillary counter-electrode voltage 5 kV; desolvation temperature 90–110°C; dry gas 6 L min^-1^; spectra collection range 800–2500 m/z. The carrier buffer was a 5:95 mixture of acetonitrile:ammonium acetate (15 mM, pH 7.0). The Cu-TmMT samples were analyzed by injecting 20 μL of protein solution at 40 μL min^-1^; capillary counter-electrode voltage 3.5 kV; lens counter-electrode voltage 4 kV; dry temperature 80°C; dry gas 6 L min^-1^. Here, the carrier was a 10:90 mixture of acetonitrile:ammonium acetate, 15 mM, pH 7.0. Acidic-MS conditions, which cause the release of the divalent metal ions from the MT complexes, but keeps the Cu(I) ions, were used to generate apo-TmMT forms and to analyze the Cu-containing samples. To this end, 20 μL of the preparation were injected under the same conditions described previously, but using a 5:95 mixture of acetonitrile:formic acid, pH 2.4, as liquid carrier. For all the ESI-MS results, the error associated with the mass measurements was always inferior to 0.1%. Masses for the holo-species were calculated as described in [34].

## Results and Discussion

### Identification and analysis of MT coding sequences in *Tremella mesenterica* databases

The discovery of the *C*. *neoformans* long MTs [16] and their relevance in pathogenesis, prompted us to analyze several fungal genomes for the presence of unusually lengthy MTs. Among all the retrieved BLAST matches using *C*. *neoformans* CnMT1 and CnMT2 as queries in the NCBI databases, the sequence derived from the *T*. *mesenterica* NCBI EIW70699 ORF (Fig 1A) was the clearest MT-like candidate, as 25% of its 104 residues were cysteine and it contained no aromatic amino acids. Nevertheless, since it constituted an incomplete protein sequence in view of its lack of a N-terminal initial methionine, the *T*. *mesenterica* genome was searched in the JCI BioProject (PRJNA32829), and its encoding sequence was localized in the TREMEscaffold_3 (access code JH711530.1). This 750 bp-long sequence was located between positions 1234899 and 1235652 in this scaffold and it included unambiguous exon/intron limits, according to the canonical splicing signals (GT/AG). In concordance with the protein sequence, this gene fragment did not show any ATG starting codon in frame, but it exhibited a stop codon (TGA) after the last serine triplet. All these observations were indicative of a 5’-truncated gene and cDNA in the *T*. *mesenterica* genome annotation, so it was necessary to further investigate the *TmMT* cDNA start.

### Determination of the full-length *T*. *mesenterica* MT cDNA and gene

**Fig 2:**
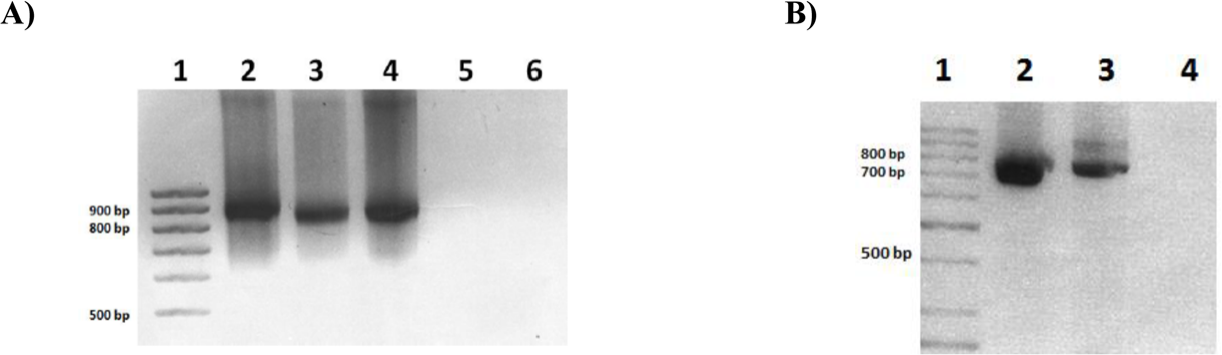
**(A) PCR products of the TmMT 5’ RACE reaction.** Lanes are: (1) DNA size markers, (2) first RACE amplification reaction, (3) second RACE amplification reaction 1:20 dilution, (4) second RACE amplification reaction, (5 and 6) negative control RACE reactions (no template DNA included). **(B) PCR products of the RT PCR and 5’ RACE TmMT amplification reactions.** Lanes are: (1) DNA size markers, (2) the PCR product of the RT-PCR reaction using TmMT specific primers, (3) final product of the RACE amplification, (4) negative control PCR reaction (no template DNA included).

**Fig 3:**
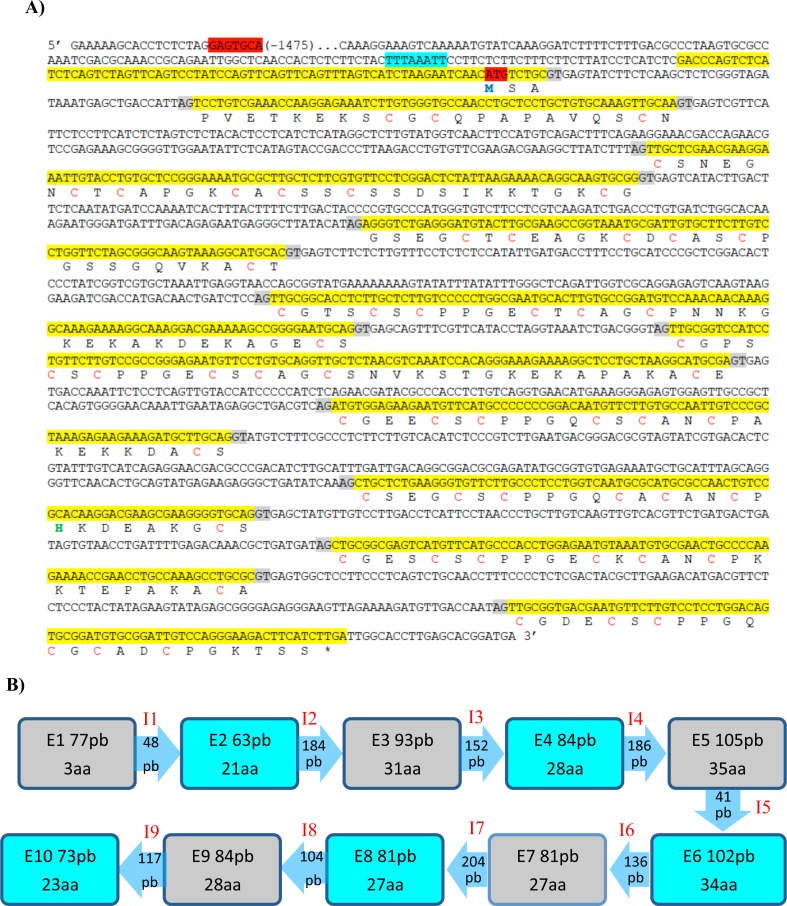
**(A) Complete TmMT cDNA and protein sequence.** The TmMT cDNA sequence obtained from the RACE reactions has been localized into the *T*. *mesenterica* genomic sequence (BioProject PRJNA32829, access code # JH711530.1), and the corresponding gene and protein significant elements have been localized. Exons are shown in yellow and the corresponding splicing donor/acceptor sites in gray, and the translational initiation codon is in red. A putative TATA is boxed in blue and a putative MRE in red. In the protein sequence, Cys are in red, His in green and Met in blue. **(B)** Scheme showing the *TmMT* gene structure (exon and intron sizes). This sequence has been submitted to GenBank and has the accession number TPA: BK008867.

The full TmMT transcript 5’ sequence was determined by RACE. Sequencing of the final RACE product (Fig 2) allowed to unambiguously identify the *TmMT* ATG start codon nearly 1200 bp upstream from the start of the truncated sequence initially retrieved (Fig 1B) from the *T*. *mesenterica* genome, and also revealed the presence of a 69- bp 5’ UTR region in the corresponding cDNA (Fig 3).

### Analysis of the *T*. *mesenterica* MT gene, cDNA and protein sequences

**Fig 4:**
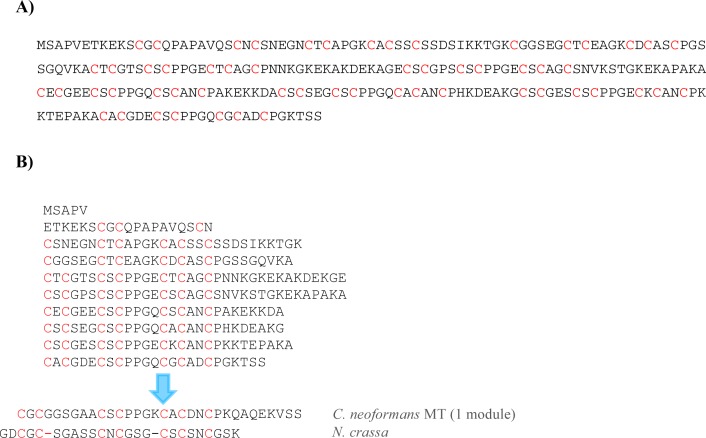
**(A) Protein sequence of TmMT and (B) proposed modular structure of TmMT**. The alignment with one 7-Cys segment of *C*. *neoformans* CnMT2 and *N*. *crassa* MT is also displayed.

The full-length *T*. *mesenterica* MT protein and cDNA sequences (Fig 3) were submitted to GenBank, and are available under the accession numbers: AJK28606.1 and KM244758.1, respectively. The TmMT cDNA coding portion (from the ATG to the STOP codons) encompasses 774 bp, and encodes a 257 aa-long protein with a molecular weight of 25.37 kDa, including 57 Cys (23% of the polypeptide) and 1 His (Fig 4A). A RT-PCR reaction on total *T*. *mesenterica* cDNA (retrotranscribed from total RNA preparation) corroborated that it was not a cloning or amplification artifact. The result of this reaction (Fig 2B) yielded a unique band of the expected size (approximately 834 bp), the sequence of which matched those of the corresponding regions in the genomic DNA database (Fig 3A). Therefore, it was established that *TmMT* was a *real* coding gene, with a sequence split into 10 exons interrupted by 9 introns (Fig 3B). The cDNA 5’ UTR had 69 bp, and an *in silico* analysis of 1500 bp of the scaffold sequence upstream the *TmMT* translation start site revealed the presence of a putative TATA box at -38 bp, an also a putative MRE at -1457 bp. It is worth noting that when the retro-PCR was carried out using RNA preparations obtained from cells grown in Cu-enriched cultures, no difference was observed in the intensity of the TmMT product, which indirectly suggests a rather constitutive gene expression pattern (data not shown).

The isolated TmMT cDNA presented ten site variations regarding the sequence in the JH11530.1 scaffold, five of which represented a missense mutation, involving the following substitutions: N22S, S35P, T48I, K201E and S207T, neither of them altering the expected metal ion coordination properties of a MT polypeptide. Overall, these substitutions (representing a 1.25% of the total coding sequence) were fully compatible with a natural polymorphism between the *T*. *mesenterica* strain used in this work and that used for genomic sequencing an annotation in the JCI project. TmMT, with 257 amino acids and 57 Cys, is the longest MT protein ever reported, longer than the *C*. *neoformans* CnMT2 isoform (183 aa), the *Tetrahymena thermophila* MTT1 isoform (162 aa) and the *Paramecium sp*. PMCd1 protein (203 aa, [35]), all of them belonging to unicellular eukaryotes. Forty-eight of its 57 Cys are organized in CXC motifs, the most extended arrangement of the Cys residues in the MT sequences, but furthermore, the analysis of TmMT readily revealed an internal repetition pattern (Fig 4B), which suggests that it was originated by a modular amplification process, as shown for *Cryptococcus neoformans* [16,17] *and Tetrahymena* [18,36] MTs. In the case of TmMT, the most obvious delimitation of hypothetical internal repetitions involves six units with a -CXCX_3_CSCPPGXCXCAXCP- sequence including 7 Cys in an arrangement alignable to that of the paradigmatic *Neurospora* MT, two units with six Cys, and a N-terminal segment with only three Cys. Other sequence features also support the homology between the TmMT building blocks and those proposed for the *C*. *neoformans* MTs, such as the occurrence of a proline doublet after the second CXC motif, a single proline after the last Cys of the segment, and the high similarity of the Cys interspersed amino acids, with a clear predominance of small residues (glycine, alanine), and the conservation of charged residues in key positions (lysine).

### The recombinant synthesis of the TmMT protein

**Fig 5:**
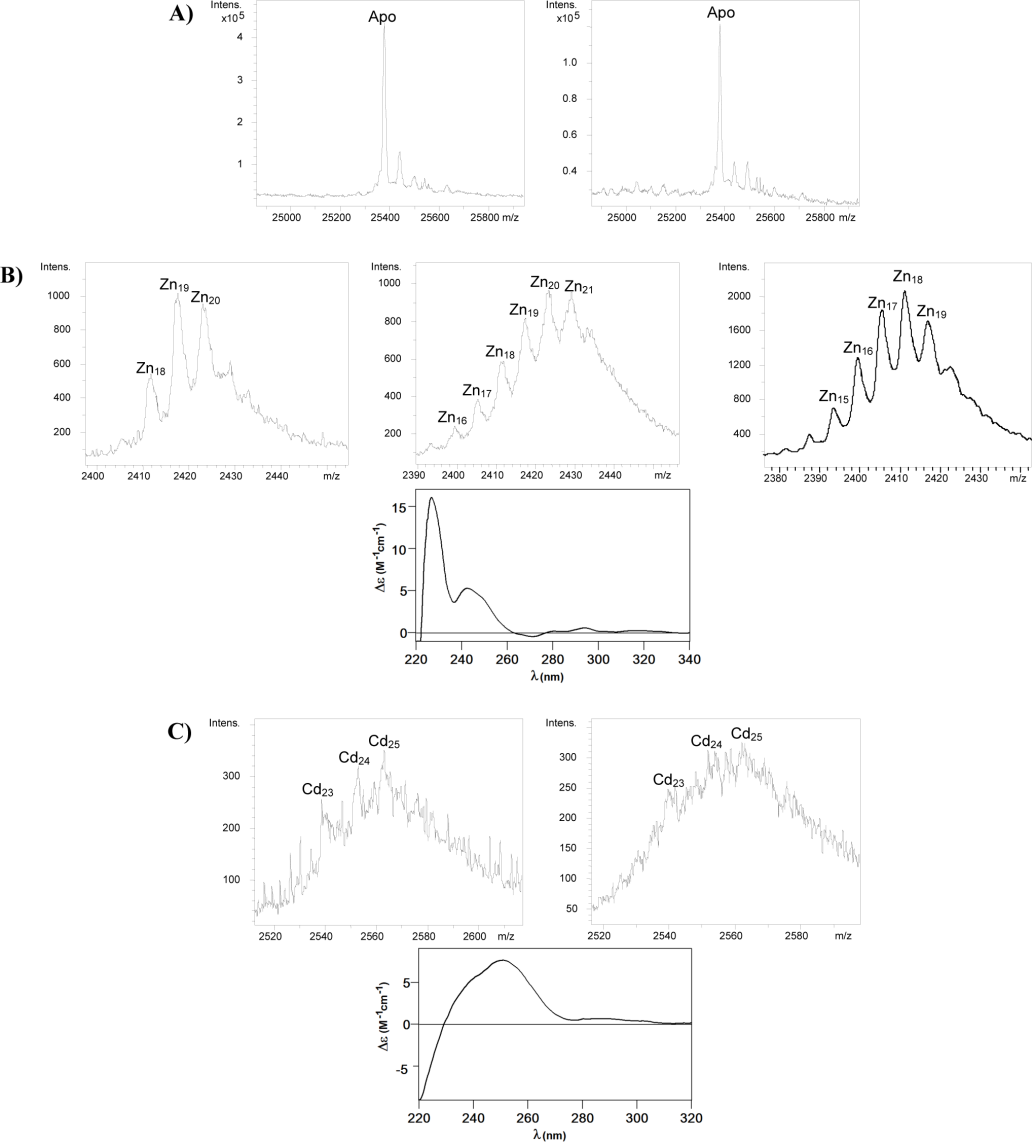
Spectroscopic and spectrometric analyses of the Zn- and Cd-TmMT preparations. (A) Deconvoluted ESI-MS spectra of the recombinant Zn- and Cd-TmMT samples, run at acid pH (2.4). A practically unique peak, corresponding to the expected molecular weight of the protein is observed in each spectrum. (B) ESI-MS spectra of three different Zn-TmMT syntheses (at the +11 charge state) recorded at neutral pH. The CD spectra corresponding to Zn-TmMT_2_ is included, those for Zn-TmMT_1_ and Zn-TmMT_3_ being completely similar. (C) ESI-MS spectra of two different Cd-TmMT syntheses (at the +11 charge state) recorded at neutral pH. The CD spectra corresponding to the Cd-TmMT_2_ is included; that for Cd-TmMT_1_ being completely similar.

DNA sequencing of several pGEX-TmMT constructs confirmed that they included no nucleotide substitutions, and that the cDNA was cloned in correct frame after the GST coding sequence. Protein extracts of small-scale (3 mL) cultures of pGEX-*TmMT*-transformed BL21 cells yielded an exclusive band a *ca*. 51 kDa, concordantly with the size of the expected fusion protein (*ca*. 26 kDa of the GST plus *ca*. 25 kDa of TmMT) (data not shown). Consequently, synthesis of TmMT was then performed in large scale cultures (5-L) supplemented with Zn(II), Cd(II) or Cu(II). Aliquots of the complexes purified from Zn- and Cd-enriched cultures (Zn-TmMT and Cd-TmMT, respectively) were first analyzed by acid (pH 2.4) ESI-MS, which revealed an almost unique peak of 25377.0 Da (Fig 5A) corresponding well with the TmMT MW of 25377.62 Da theoretically calculated for the recombinant peptide, including N-terminal Gly and Ser residues derived from the GST-fusion construct. Thus, the identity, purity and integrity of the recombinant TmMT were fully confirmed.

### The divalent metal ion (Zn(II) and Cd(II)) binding abilities of TmMT

The recombinant synthesis of TmMT in cells grown under Zn-supplementation yielded a mixture of Zn-complexes of different stoichiometries, with Zn_17_- to Zn_21_-TmMT as major species (Table 2 and S1 Table, and Fig 5B). This matched well with the Zn-per-protein mean content calculated by ICP-AES, which gave values between 14.0 and 20.1 (Table 2). The CD spectra of the three samples preparations were practically identical, exhibiting a Gaussian band centered at *ca*. 240 nm typical of the Zn-Cys chromophores (Fig 5B).

**Table 2:** Recombinant synthesis yield and metal-to-protein ratios of the purified Zn-, Cd- and Cu-TmMT complexes, according to neutral ICP-AES and ESI-MS measurements.

Metal-TmMT syntheses^a^	[TmMT] (10^−4^ M)	ICP-AES^b^			Neutral ESI-MS^c^				Acid ESI-MS^c^
		Zn/MT	Cd/MT	Cu/MT	Zn species	Cd species	Zn/Cu species	Cu species	Cu species
**Zn-TmMT**_**1**_	0.71	20.1			Zn_21_				
					**Zn**_**20**_				
					**Zn**_**19**_				
					Zn_18_				
**Zn-TmMT**_**2**_	0.74	14.0			**Zn**_**21**_				
					**Zn**_**20**_				
					Zn_19_				
					Zn_18_				
					Zn_17_				
					Zn_16_				
**Zn-TmMT**_**3**_	0.94	16.8			Zn_19_				
					**Zn**_**18**_				
					**Zn**_**17**_				
					Zn_16_				
					Zn_15_				
**Cd-TmMT**_**1**_	0.54		24.2			**Cd**_**25**_			
						Cd_24_			
						Cd_23_			
						multiple			
**Cd-TmMT**_**2**_	0.62		24.5			**Cd**_**25**_			
						Cd_24_			
						Cd_23_			
						multiple			
**Cu-TmMT (normal aeration)**	0.55	6.6		5.8			**M**_**15**_		Cu_12_
							M_19_-M_8_		Cu_10_
									Cu_9_
									**Cu**_**8**_
									Cu_6_
									Cu_5_
									**Cu**_**4**_
									Apo
**Cu-TmMT (low aeration)**	0.05			40.0				**Cu**_**42**_	**Cu**_**38**_
								**Cu**_**41**_	**Cu**_**36**_
								Cu_45_-Cu_38_	Cu_38_-Cu_29_

^a^ Several syntheses were performed in each metal supplemented medium, which are numbered by subscripts.

^b^ In all cases, the Zn, Cd, Cu and S content was measured by ICP-AES, but only the detectable contents are shown. Protein concentration was calculated from the S content in normal ICP-AES measurements.

^c^ Stoichiometries were calculated from the mass difference between the holo- and apo-proteins. Major species are in bold. M = Zn or Cu which have a molecular mass indiscernible by ESI-MS. *Multiple* means that a continuum of peaks are observed between the indicated values.

The biosynthesis of TmMT in Cd-supplemented cultures revealed its poor ability for coordination of this metal ion, even lower than for Zn(II). The synthesis was repeated several times, and practically always it was impossible to discriminate individual peaks (Table 2 and S1 Table, and Fig 5C). The only data that can be suggested from the ESI-MS spectra was that most probably the major complexes were those including between 23 and 25 Cd(II), this coinciding with results of ICP-AES that yielded an average content of 24.2 to 24.5 Cd(II) per TmMT (Table 2). The CD spectrum showed the typical fingerprint attributable to tetrahedral Cd(SCys)_4_ chromophores absorbing at *ca*. 250 nm, and a slight absorption in the region of 280–300 nm, probably contributed by the presence of some Cd-S^2-^ chromophores (Fig 5C). Overall, all the previous results lead to the conclusion that TmMT is far from exhibiting a binding preference for divalent (Zn(II) or Cd(II) [8]). This is reflected in the formation of a continuum of species, none of them energetically favored, with both Zn(II) and Cd(II) ions, and also accounts for the lack of reproducibility of the recombinant syntheses, which is patent in this work for the Zn-complexes, and which has been observed also before, among others, for the *Tetrahymena pyriformis* MT1 [37] and *C*. *neoformans* MT1 isoforms [17]. The presence of S^2-^ ligands in the Cd-TmMT complexes also corroborates this inability [33].

### Cu-binding abilities of TmMT

**Fig 6:**
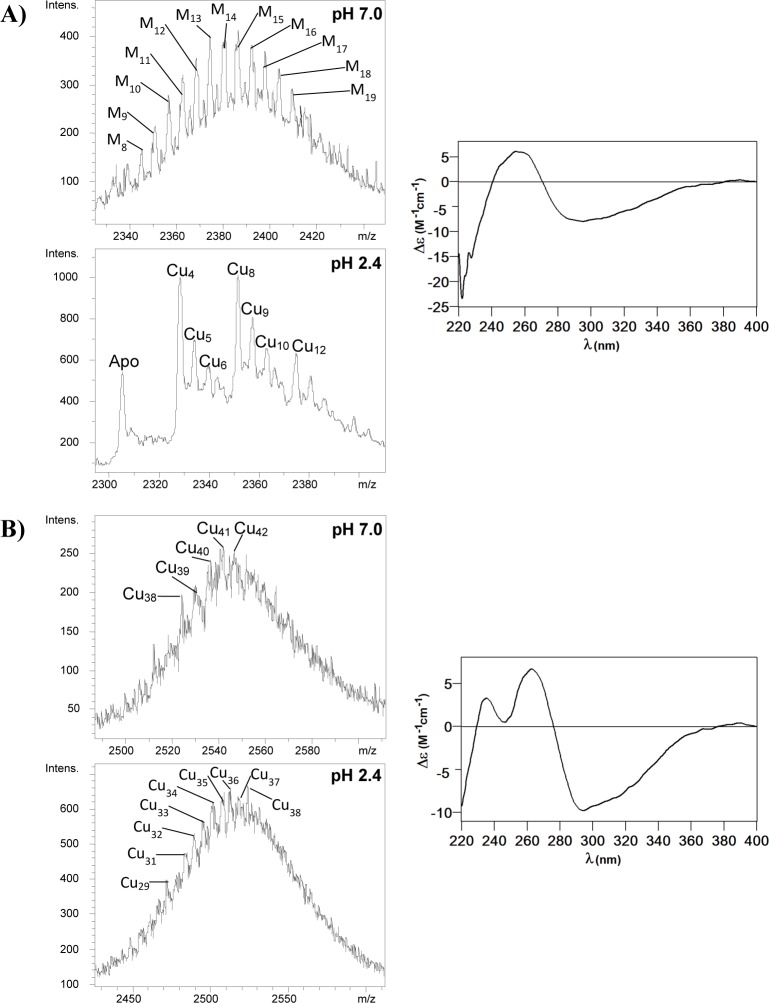
Spectroscopic and spectrometric analyses of the Cu-TmMT preparations. (A) ESI-MS spectra of the recombinant Cu-TmMT synthesized in normally-aerated cultures (at the +11 charge state), run at neutral pH (7.0) and acid pH (2.4), and CD spectrum of the same sample. (B) ESI-MS spectra of the Cu-TmMT synthesized in low-aerated cultures (at the +11 charge state), run at neutral pH (7.0) and acid pH (2.4), and CD spectrum of the same sample.

TmMT was recombinantly synthesized in Cu(II)-supplemented cultures grown under normal and low aeration, because this modulates the amount of Cu available inside the host cells for recombinant Cu-MT complex formation [29]. Hence, when TmMT was synthesized in normally aerated cultures, it yielded a mixture of Zn,Cu-TmMT species ranging from M_8_- to M_19_-TmMT (M = Zn+Cu), with those from M_12_- to M_17_-TmMT as major products, as shown by ESI-MS at neutral pH (Table 2 and S2 Table, and Fig 6A). ESI-MS at pH 2.4 revealed that this continuum was formed by heterometallic complexes with Cu(I) contents showing a peculiar periodicity: multiple of 4, and multiple of 4 plus one, that is: Cu_4_ and Cu_5_, Cu_8_ and Cu_9_, and Cu_12_ and Cu_13_ (*cf*. Table 2 and S2 Table, and Fig 6A). In contrast, low aeration yielded a mixture of homometallic Cu-TmMT species -since ICP-AES measurements only detected this metal-, which ranged between Cu_36_- and Cu_45_-TmMT, with major species being Cu_42_- and Cu_41_-TmMT (Table 2 and S2 Table, Fig 6B). Acid ESI-MS of this sample revealed a similar distribution of species, of slightly lower stoichiometry (Cu_29_ and Cu_38_) probably attributable to some loosely bound Cu(I) ions. Interestingly, both types of preparations rendered well defined and closely related CD spectra (Fig 6A and 6B). The CD fingerprint of the sample purified from normally aerated bacteria showed a wide absorption between 240 and 270 nm, while that of the low-aerated productions precisely showed two maxima with no absorption at 240 nm, which reinforces the presence of Zn(II) ions in the former.

**Fig 7:**
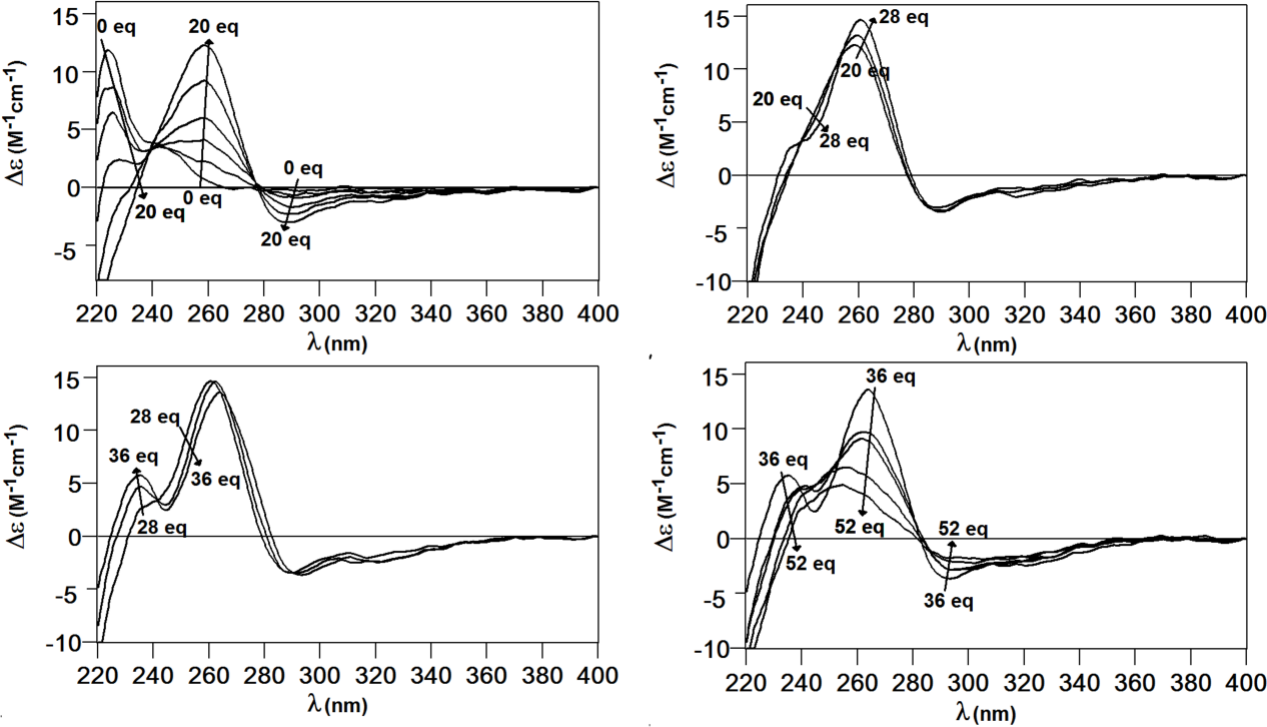
CD spectrum of the Zn/Cu exchange reaction in Zn-TmMT. The CD spectra were recorded every 4 Cu(I) eq added to a 3 μM solution of Zn-TmMT.

**Fig 8:**
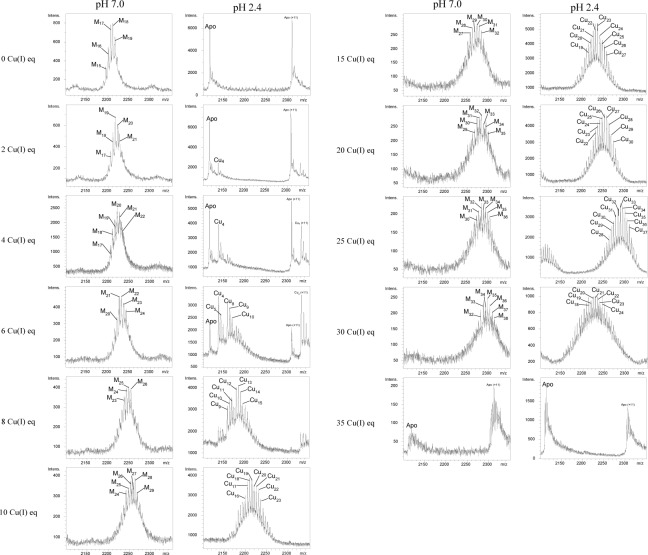
ESI-MS spectra of the Zn/Cu exchange reaction in Zn-TmMT. The ESI-MS spectra were measured, both at pH 7.0 and pH 2.4, on aliquots of a 94 μM solution of TmMT every 2 Cu(I) eq added at the beginning of the reaction and every 5 Cu(I) between 10 and 35 equivalents added.

Most informative results about TmMT Cu(I) binding abilities came from the study of the species constituted *in vitro* by Zn/Cu exchange. Nevertheless, these experiments revealed an unprecedented feature regarding the standard methodological procedure used [31], because spectroscopic (CD, UV-vis) and spectrometric (ESI-MS) measurements could not be performed on the same sample due to incompatible concentration requirements. Thus the CD and UV-vis data was collected on a 3 μM solution of Zn-TmMT with additions of 4 Cu(I) eq each to avoid detector saturation (Fig 7) and the ESI-MS data on a *ca*. 100 μM solution, which enabled the detection of the high number of species present in solution (Fig 8). The CD spectra of the successive reaction steps draw very neat isodichroic points, which strongly suggested a cooperative copper loading and zinc displacement process. These isodichroic points arise by the decrease of the *ca*. 230 nm band and the increase of the 260(+) and 290(-) nm CD absorptions, owing to the creation of Cu-SCys chromophores from 0 to 28 Cu(I) eq added. After a small rearrangement between 28 and 36 Cu(I) eq, further additions provoke the collapse of the CD signal.

It is evident that, from the beginning, the addition of Cu(I) enhances the already high complexity of the initial Zn-TmMT sample, generating a larger number of species in solution (Fig 8). The heterometallic species formed increase in nuclearity up to a maximum of M_38_-TmMT (M = Zn or Cu), after the addition of 30 Cu(I) eq, which is the step yielding one of the most intense CD spectra (Fig 7). However, the most remarkable hints about the replacement reaction are revealed by the acid ESI-MS analyses, because they suggest how TmMT builds its copper complexes. Hence, the addition of just 4 Cu(I) eq at the beginning of the experiment already gave rise to a predominant Cu_4_-core, accompanied by a minor Cu_5_-cluster, and species of higher nuclearity which will become significant later. Thence, at the following step, Cu_4_- and Cu_5_-TmMT are still significant, but the doublet Cu_8_- and Cu_9_- is almost as important. In the next step, Cu_4_-, Cu_5_- and Cu_6_- Cu_8_- have practically disappeared, and the predominant cores are the Cu_12_- and Cu_13_-clusters. From this point on (*i*.*e*. 10 Cu(I) eq added), the complexity of the Cu(I)-containing species is very high, with a continuum of Cu(I)-cores, the Cu(I) content of which increases until approximately 40. It is worthwhile noting that the addition of 25 Cu(I) eq was necessary to totally displace the Zn(II) initially bound to TmMT. Finally, in the presence of an excess of copper beyond 30 Cu(I) eq added, the Cu-TmMT complexes become unstable, so that the Cu(I) nuclearity of the detected species decreases, and at the end of the reaction (Cu overload conditions) only the apo-TmMT peptide is detected, which has been reported also for other Cu-thioneins [38,39]. From these data, a certain periodicity of the predominant Cu(I) clusters can be deduced, so that in the two first steps, they were Cu_4_-units always accompanied by a more or less important peak with an additional Cu(I) (*i*.*e*. Cu_4_- and Cu_5_-, and then Cu_8_- and Cu_9_), and from here onwards, the following predominant peaks result from adding 5 Cu(I) to the previous species (*i*.*e*. Cu_12_- and Cu_13_-; Cu_17_- and Cu_18_-; Cu_22_- and Cu_23_-; Cu_27_- and Cu_28_- and Cu_33_-TmMT). Although a strict cooperative process for the Zn/Cu displacement in Zn-TmMT has to be ruled out owing to the many different species coexisting during the experiment, a clear periodicity for the major Cu(I) content of created species is observed, which can be related to the modular structure of the TmMT polypeptide (*cf*. Fig 4). Characterization of the Cu-binding abilities of the also modular *C*. *neoformans* CnMTs showed that their 7-Cys boxes render Cu_5_-clusters of additive behavior [16,17,26], and coincidently the TmMT results suggest that its 7-Cys stretches may exhibit a similar behavior. Additionally, TmMT has two 6-Cys boxes, which can be assumed to optimally bind 4 Cu(I) ions. The remaining 3 Cys would also contribute to bind extra Cu(I) ions. This will explain the series observed for the Cu(I)-core composition in the Zn/Cu exchange reaction. Precisely, both [Cu_4_S_6_] and [Cu_5_S_7_] clusters were characterized by classic inorganic chemistry model studies as the most relevant flexible cores in relation to Cu-MT complexes [40,41,42].

Finally, it is worth noting that, also as first noticed for the yeast Crs5 MT [29] and later reported for other Cu-thioneins, the results of TmMT synthesis in Cu-supplemented media at both assayed aerations is reproduced in two different steps of the Zn/Cu exchange process, at least in relation to the Cu(I) cores present. Hence, the results of the Cu-TmMT production in regularly aerated cultures correspond to the Zn-TmMT+6 Cu(I) eq added stage (*cf*. CD features and ESI-MS spectra in Figs 6, 7 and 8), while the results from low aerated syntheses correspond to the last steps of the titration, just before the unfolding of the Cu-TmMT complexes caused by an excess of Cu(I) ions (*cf*. CD features and ESI-MS spectra in Figs 6, 7 and 8). This indicates that the results of these recombinant syntheses effectively correspond to two different situations of Cu-availability.

### Copper tolerance of native *Tremella mesenterica* and *Saccharomyces cerevisiae* cells transformed with TmMT

**Fig 9:**
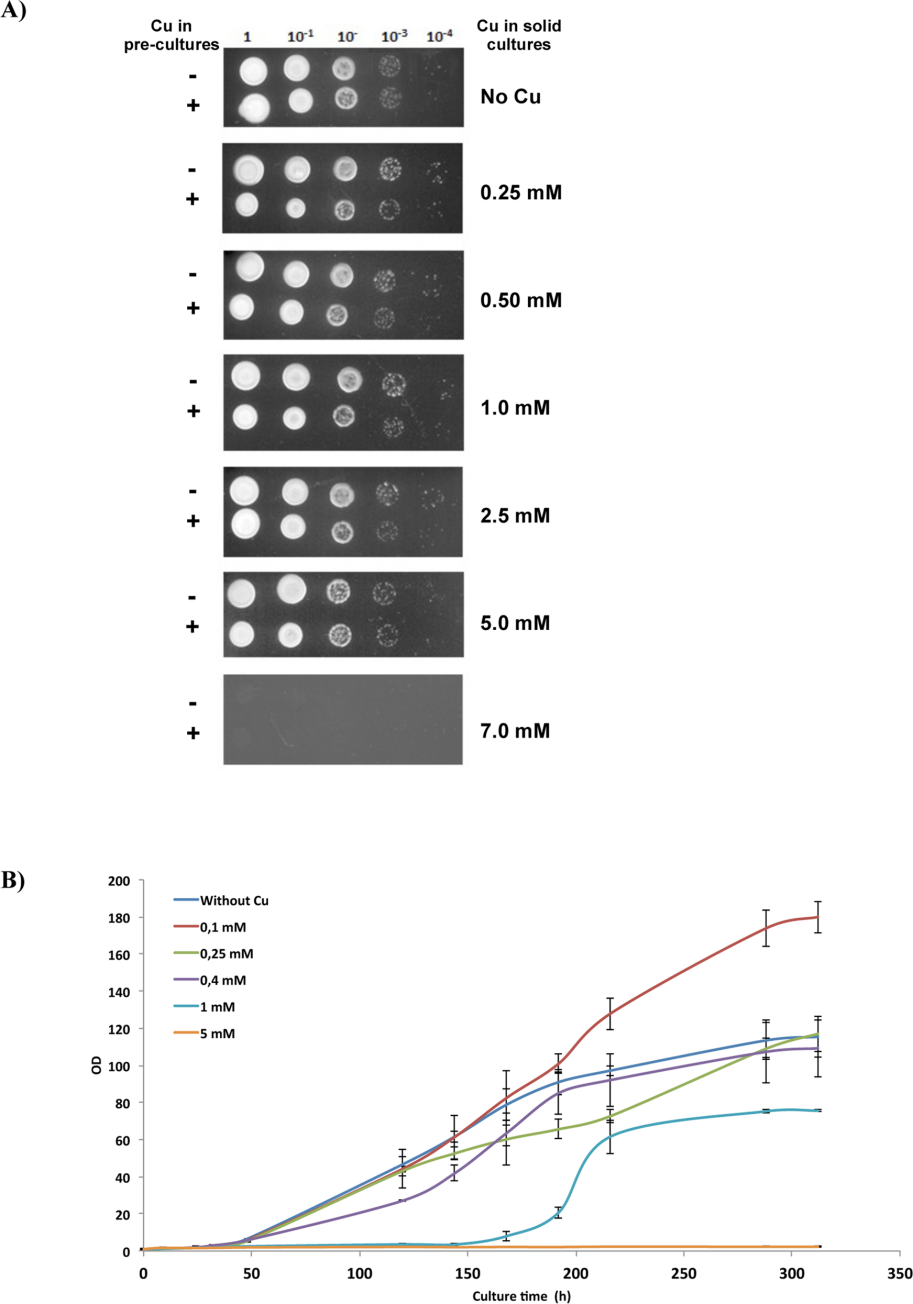
*Tremella mesenterica* growth under Cu supplementation conditions. (A) *T*. *mesenterica* solid cultures. Pre-cultures were grown in YPD liquid medium overnight at 25°C (first row in each condition without added Cu and second row, with 0.1 mM Cu added), then diluted to OD_600_ 0.5 and spotted into the YPD agar plates at 4 serial dilutions. Plates were allowed to grow at 30°C for 3 days. For more details, see the Experimental Procedures section. (B) Growth curve representing the normalized mean values of the OD_600_ exhibited by the liquid cultures grown in MY medium supplemented with the indicated Cu concentrations. Results represent the mean and standard deviation (vertical bars) of at least three replicates.

*T*. *mesenterica* copper tolerance assayed in solid cultures revealed a rather unaltered growth up to 5.0 mM Cu, and at 7.0 mM Cu cells were unable to grow. In this experiment, each Cu concentration was assayed in two conditions, depending on whether the pre-culture spotted on the plates had been supplemented with Cu (0.1 mM) or not (Fig 9A). No significant differences could be observed. When Cu tolerance was measured in liquid cultures, two different phases were observed in long-term cultures (up to 300 h), taking into account that this fungus exhibits a slow growth, and a clear increase in the OD_600_ was not observed until 50 h after inoculation (Fig 9B). Hence, until 150 h growth, all the cultures grew at an equivalent rate, except those supplemented with 1 mM Cu, which exhibited a lengthened lag phase in concordance with the stress imposed by the excess of Cu. All these results confirm a high Cu tolerance for this fungus, comparable to that of the CUP1 multicopy *S*. *cerevisiae* strains [43], considering that only one copy of the *TmMT* gene was detected in the genome by the *in silico* screening. However, it has to be noted that this increased tolerance is manifested in lasting cultures, maybe in relation to the slow *T*. *mesenterica* growth dynamics. Noteworthy, the culture supplemented with 0.1 mM Cu exhibited a maintained increased growth in relation to all other conditions, so that it could be hypothesized that the optimal growth of *T*. *mesenterica* depends on the presence of this metal ion in its natural environment.

**Fig 10:**
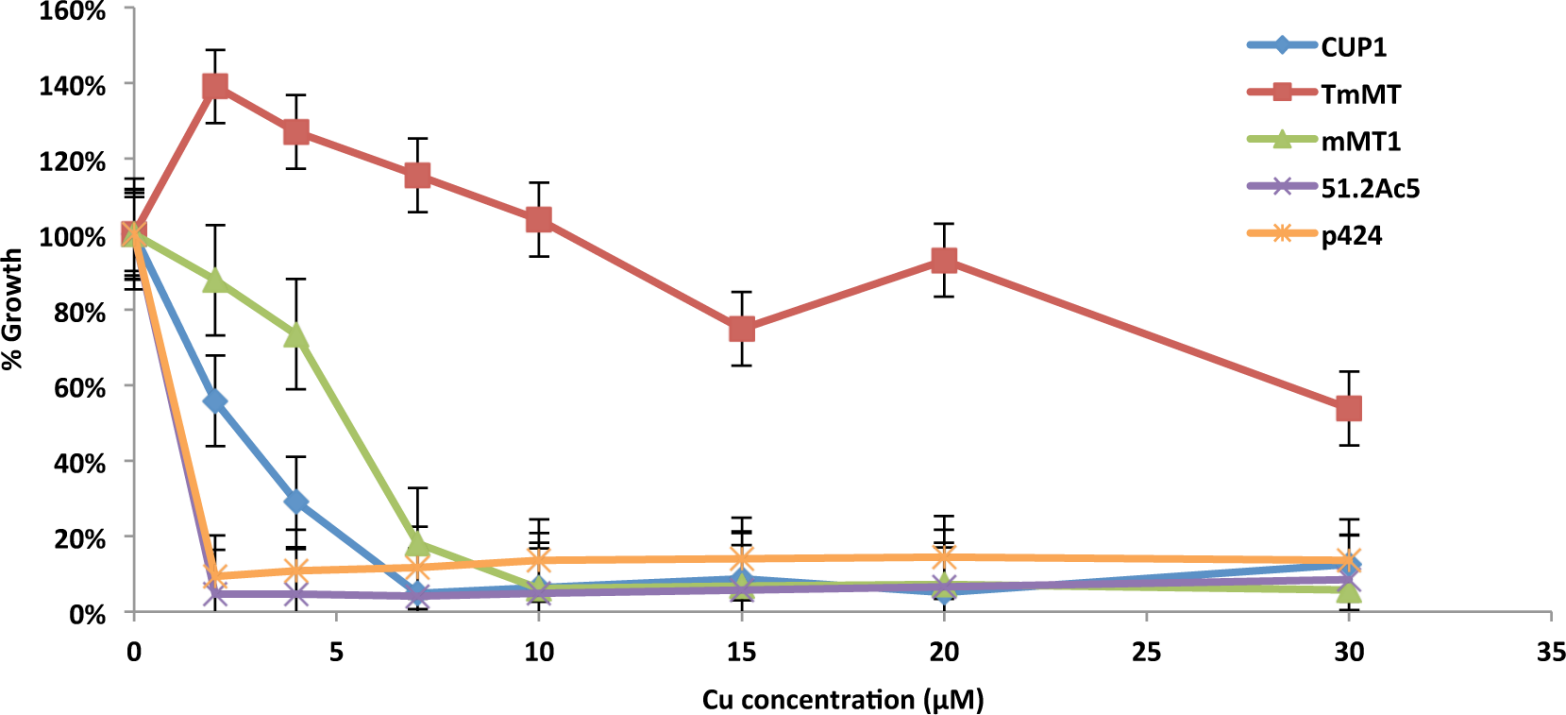
Effect of the heterologous expression of the *T*. *mesenterica* TmMT in *S*. *cerevisiae* 51.2cΔc5 MT-null strain. Yeast cells were transformed with the different p424-MT constructs. Besides p424-TmMT, cells transformed with the void p424, and the p424-CUP1 (the yeast MT) and p424-mMT1 (the mammalian isoform MT1) plasmids were also included in the assay for comparative purposes. 3-mL cultures grown overnight at 30°C were diluted to OD_600_ 0.1 in 3-mL fresh SC-Trp-Ura medium supplemented with 0, 2, 4, 7, 10, 15, 20, and 30 μM CuSO_4_ and grown for 18h at 30°C. The growth of the host 51.2cΔc5 strain cultured in complete medium was also assayed. Growth was evaluated in liquid cultures and it is represented as the percentage of the growth rate attained in a non-Cu supplemented medium. All the experiments were carried out at least by triplicate and the plotted points represent the mean value for each condition associated to its corresponding error bar. The data unambiguously show the extraordinary copper tolerance conferred by TmMT. For more details, see the Experimental Procedures section.

To confirm the ability of the *TmMT* to confer high-Cu resistance, the effect of its heterologous expression in the *S*. *cerevisiae* 51-2c-Δc5 strain, which lacks its two MT genes (*CUP1* and *CRS5*) was assayed. The ability of the cells transformed with *TmMT*-p424, to grow in liquid media supplemented with increasing copper concentrations was represented as percentage of the growth exhibited in cultures with no Cu supplementation (Fig 10). For comparative purposes, the *CUP1*-p424 and *mMT1*-p424 constructs, containing respectively the *S*. *cerevisiae* and the mouse MT1 isoforms, as well as the void p424 plasmids were also assayed. The results unambiguously show the extraordinary tolerance to Cu conferred by the expression of *TmMT* in yeast cells, in all the assayed Cu concentrations, up to 30 μM. What is more, it allows cell survival, and at considerable levels, beyond 10 μM Cu, when expression of either *CUP1* or *mMT1* fails to maintain cell growth (Fig 10).

## Conclusions

One gene encoding a metallothionein polypeptide is present in the *Tremella mesenterica* genome, hereinafter called, *TmMT*. It has been identified *in silico* from a partial protein sequence found in a *T*. *mesenterica* data bank as starting query, and also from total mRNA retrotranscription and specific amplification, which has confirmed that the predicted *TmMT* ORF was a *real* coding sequence of uncommon length for a *MT* gene. The *TmMT* gene covers more than 2 kb of DNA, and its coding sequence is separated in 10 small exons -sizes from 63 bp to 105 bp-, interrupted by 9 introns, from 48 bp to 204 bp. The 5’ UTR of this cDNA is 69-bp long, and in the upstream gene sequence, a putative TATA box and an MRE element have been identified. The cDNA coding portion encompasses 774 bp, and the resulting protein has 257 amino acids, 57 of which are Cys distributed in CXXC motifs as corresponding to the common hallmarks of Metallothioneins. Therefore, this is the longest MT polypeptide reported up to now, beyond the *Paramecium sp*. PMCd1 protein (203 aa) the fungal *C*. *neoformans* CnMT2 (183 aa) and CnMT1 (122 aa) isoforms. Coincidently with the *C*. *neoformans* MTs, the TmMT polypeptide exhibits clear internal repetition patterns, which suggests that modular amplification processes of a primeval fungal MT -represented by the paradigmatic short MTs reported in the *Neurospora* or *Agaricus* genus- would be in the bases of the genesis of long MTs in the Tremellales order of Ascomycota fungi. In the case of TmMT, the most obvious arrangement of hypothetical internal repetitions is the alignment shown in Fig 4B, for which six of the units depict a -CXCX_3_CSCPPGXCXCAXCP- motif including 7 Cys, as for paradigmatic fungal MTs (*i*.*e*. *Neurospora* MT), and includes two units with 6 Cys and a N-terminal segment with only 3 Cys.

The analysis of the metal binding abilities of TmMT confirmed that it exhibits all the features of a high-capacity Cu-thionein. Hence, when TmMT coordinates Zn(II) ions, it renders a mixture of species of different stoichiometries, which is also the case for Cd(II) coordination. This patent inability for divalent metal ion coordination is accompanied by a clear behavior of Cu-thionein, although its large size entails that when synthesized in regular Cu concentrations, the obtained complexes are heterometallic (Zn, Cu) species, just as the *C*. *neoformans* CnMT1 and CnMT2 were. However, at high Cu concentrations (*i*.*e*. produced in host cells grown under poor aeration), TmMT is able to render homometallic complexes of as high nuclearity as 45 Cu(I), with the major species being Cu_40_ to Cu_42_-TmMT. The modular composition reported for the *C*. *neoformans* Cu-CnMT complexes is probably also re-encountered in Cu-TmMT, since its polypeptide sequence is easily dissected in alignable stretches: six 7-Cys boxes, two 6-Cys boxes, and three N-terminal Cys. This assumption would also explain the Cu contents of the most favored Cu-species. Strong similarity is also observed for some residues located between the coordinating Cys (*i*.*e*. the proline doublet after the second CXC motif, a single proline after the last Cys of the segment, a clear predominance of small (glycine, alanine) residues, and the conservation of charged residues in other key positions (lysine)). Thus, it is tempting to associate these features, not only to the evolutionary origin and conservation of this protein pattern, but also to their optimization for Cu(I) coordination geometry.

Of course, the most interesting question raised by these results concerns the physiological role that this MT protein may play in its native surrounding. A first approach is to consider the natural habitat where *Tremella* grows, mainly decaying wood, suggests that Cu-handling may be of the utmost importance to provide active lignin-metabolizing enzymes, such as laccases and peroxidases, which are well-known Cu-containing oxidases. This would be in concordance with the constitutive, rather than inducible, *TmMT* pattern expression, since it could be considered a housekeeping gene functionality. Appealingly, this hypothesis might, in the end, link the biological function of TmMT with that proposed for the Cu-MTs of the pulmonate snails, which are supposed to serve as storage/chaperones of Cu for the hemocyanins, the respiratory O_2_ carriers of these organisms [44].

## Supporting Information

S1 Table Experimental molecular masses (ESI-MS results) and calculated molecular masses for Zn-TmMT and Cd-TmMT species. Subindexes describe different syntheses. Major species are in bold.
(PDF)

S2 Table Experimental molecular masses (ESI-MS results) and calculated molecular masses for Cu-TmMT species synthesized in regular and low-aerated *E*. *coli* cultures. Major species are in bold. M = Zn or Cu.
(PDF)
