# Supplementary material for: The Fungus Tremella mesenterica Encodes the Longest Metallothionein Currently Known: Gene, Protein and Metal Binding Characterization
Source: PLoS One. 2016 Feb 16;11(2):e0148651. doi: 10.1371/journal.pone.0148651 (PMC4755600; doi:10.1371/journal.pone.0148651)
Supplement: S1 Table — Subindexes describe different syntheses. Major species are in bold. (PDF) [file pone.0148651.s001.pdf]

S1 Table. Experimental molecular masses (ESI-MS results) and calculated molecular masses for Zn-TmMT and Cd-TmMT species. Subindexes describe different syntheses. Major species are in bold.

Synthesis (metal supplemented)	ESI-MS pH	Identified species	Experimental mass (Da)	Theoretical mass (Da)
$Zn\text{-}TmMT_1$	7.0	Zn_{21}	26708	26707.8
		**Zn_{20}**	**26644**	**26645.4**
		**Zn_{19}**	**26578**	**26582.0**
		Zn_{18}	26515	26518.6
	2.4	apo	25377	25377.6
$Zn\text{-}TmMT_2$	7.0	**Zn_{21}**	**26711**	**26707.8**
		**Zn_{20}**	**26646**	**26645.4**
		Zn_{19}	26575	26582.0
		Zn_{18}	26514	26518.6
		Zn_{17}	26448	26455.2
		Zn_{16}	26384	26391.9
	2.4	apo	25375	25377.6
$Zn\text{-}TmMT_3$	7.0	Zn_{19}	26570	26582.0
		**Zn_{18}**	**26513**	**26518.6**
		**Zn_{17}**	**26448**	**26455.2**
		Zn_{16}	26384	26391.9
		Zn_{15}	26322	26328.6
	2.4	apo	25375	25377.6
$Cd\text{-}TmMT_1$	7.0	**Cd_{25}**	**28136**	**28137.9**
		Cd_{24}	28025	28026.6
		Cd_{23}	27915	27916.2
	2.4	apo	25376	25377.6
$Cd\text{-}TmMT_2$	7.0	**Cd_{25}**	**28136**	**28137.9**
		Cd_{24}	28025	28026.6
		Cd_{23}	27915	27916.2
	2.4	apo	25377	25377.6
