# Supplementary material for: The Fungus Tremella mesenterica Encodes the Longest Metallothionein Currently Known: Gene, Protein and Metal Binding Characterization
Source: PLoS One. 2016 Feb 16;11(2):e0148651. doi: 10.1371/journal.pone.0148651 (PMC4755600; doi:10.1371/journal.pone.0148651)
Supplement: S2 Table — Major species are in bold. M = Zn or Cu. (PDF) [file pone.0148651.s002.pdf]

Table S2. Experimental molecular masses (ESI-MS results) and calculated molecular masses for Cu-TmMT species synthesized in regular and low-aerated *E. coli* cultures. Major species are in bold. M= Zn or Cu.

Synthesis (metal supplemented)	ESI-MS pH	Identified species	Experimental mass (Da)	Theoretical mass (Da)
Cu-TmMT (regular aeration)	7.0	M_{19}	26557	26564.5
		M_{18}	26493	26502.0
		M_{17}	26428	26440.9
		M_{16}	26363	26378.4
		**M_{15}**	**26302**	**26315.8**
		M_{14}	26238	26253.3
		M_{13}	26176	26190.7
		M_{12}	26104	26128.1
		M_{11}	26046	26065.6
		M_{10}	25978	26003.0
		M_{9}	25907	25940.5
		M_{8}	25845	25877.9
	2.4	Cu_{12}	26107	26128.1
		Cu_{10}	25971	26003.0
		Cu_{9}	25917	25940.5
		**Cu_{8}**	**25853**	**25877.9**
		Cu_{6}	25728	25752.9
		Cu_{5}	25665	25690.3
		**Cu_{4}**	**25600**	**25627.8**
		apo	25347	25377.6
Cu-TmMT (low aeration)	7.0	Cu_{45}	28182	28192.2
		Cu_{44}	28138	28129.6
		Cu_{43}	28061	28067.1
		**Cu_{42}**	**28006**	**28004.6**
		**Cu_{41}**	**27936**	**27942.0**
		Cu_{40}	27874	27879.5
		Cu_{39}	27819	27816.9
		Cu_{38}		
	2.4	**Cu_{38}**		
		Cu_{37}		
		**Cu_{36}**		
		Cu_{35}		
		Cu_{34}		
		Cu_{33}		
		Cu_{32}		
		Cu_{31}		
		Cu_{30}		
		Cu_{29}		
